# Transactions of the Chicago Society of Physicians and Surgeons, March 9th, 1874

**Published:** 1874-03-15

**Authors:** 


### TRANSACTIONS OF THE CHICAGO SOCIETY OF PHYSICIANS AND SURGEONS.

### MEETING OF MARCH 9TH, 1874.

THE Society met as usual, in the parlor of the Grand Pacific Hotel, the President in the chair.

The minutes of the preceding meeting were read and approved.

Dr. R. H. Bingham, of Castleton Medical College, N. Y., was unanimously elected to membership; and the name of Dr. C. T. Parkes, of Rush Medical College, presented by Dr. Owens as a candidate.

Dr. W. C. Lyman then read an "Abstract of Cases Treated in the Woman's Hospital of the State of Illinois, during the year 1873." Typical cases were reported of several forms of diseases peculiar to women, and these selected from those long under consecutive treatment, in order that the tabulated results might be trustworthy. Five cases of subinvolution of the uterus were cited, in which the period which had elapsed since parturition, varied from three months to six years; ages, from twenty-three to thirty-eight years; average age, twenty-nine years; duration of treatment, two to twenty months; average duration of treatment, five months; number of local applications, four to fifty-nine; average number of local applications, eighteen. The treatment consisted, generally, of the application of nitric acid to the cervical and uterine cavities, followed by glycerine and cotton-ball pessaries, saturated with the same, kept in contact with the parts for twenty-four hours. These applications were followed by some pain, and slight hæmorrhage, readily yielding, however, to treatment by the recumbent position only. Other applications used were, saturated solution of tannin, in glycerine; Lugol's solution of iodine and glycerine; and solutions of the muriated tincture of iron.

Twenty-six cases of endometritis were next referred to, in which lesions existed of both fundus and cervix. In six of these, there was also ulceration or erosion of the margins of the os. Of the entire number, sixteen were married, and ten single; ages, from twenty-one to forty-three years: average, thirty-one; number of applications, from six to forty-eight: average, twenty-one; duration of treatment, one to twelve months: average, somewhat more than one month. Treatment, generally, by local applications of Churchill's tincture of iodine; solution of tannin, in glycerine; of the nitrate of silver, etc.; internally, mineral acids, vegetable bitters, and ferruginous preparations.

Seven cases of abnormal position of the womb were then considered, the larger number of which were treated without mechanical support, the key to the difficulty having been found in the relief of the inflammatory symptoms. Of these, five were married, and two single. Average duration of treatment, three and one-half months; average number of applications, sixteen; average age, twenty-four years. One of these cases was relieved by the application of a solution of argenti nitras, one-half drachm to the fluid ounce, introduced twice a week into the cervical cavity. Another was treated satisfactorily by the wearing of Hodge's closed lever pessary.

In all cases, special indications were promptly met.

Dr. P. S. Hayes then read that portion of the Annual Report of the Section on Pathology having regard to the nervous system. The Report was exhaustive of such material as had been published during the preceding year, on the pathology of nervous diseases. On motion, the Report was accepted.

Dr. John E. Owens then, by request of several members, made a verbal report of the operation of ovariotomy, recently done by himself, at St. Luke's Hospiral. The patient was a woman, over fifty years of age, and multiparous; and the abdominal tumor was as large as that of a pregnant female at the eighth month. Anæsthesia was induced by chloroform and ether, and the thin abdominal walls incised, without loss of blood, to a distance of six or seven inches in the mesial line, below the umbilicus. The tumor was exposed, and, as no attachments were discovered, it was readily turned out from the abdominal cavity. A single cyst, which ruptured during this process, discharged its contents into a basin held for that purpose. The tumor was found to consist of a mass of colloid material resembling, in consistency and appearance, calves'-foot jelly; and a similar degeneraton was discovered in the vermiform appendix, which was enlarged six-fold. The external surface of the small intestines was covered with red granulations, in the vicinity of the tumor, and argued ill for the success of the operation. The pedicle was secured by a clamp—an opening left for drainage—and, on the tenth day, the wound had united, and the general condition of the patient found to be as favorable as could be expected.

The speaker also read details of the following cases, occurring in St. Luke's Hospital:

CASE I.—GASO-PURULENT ABSCESS IN THE ABDOMINAL WALLS.—Sept. 17th, 1873, an unmarried girl, nineteen years of age, was admitted, with specific vaginitis. A diffused, inflammatory induration appeared, subsequently, in the abdominal walls, which was so far relieved that she left the hospital, but returned, in three and a half months, with aggravation of her symptoms, and inflammatory fever. Subsequently, a gaso-purulent abscess formed, which was left to open spontaneously. It burst in ten days after the re-admission, giving exit to a large quantity of fœtid pus and sulphuretted hydrogen gas. Convalescence was rapid; and the patient was discharged Dec. 23d, 1873.

CASE II.—PARACENTESIS, AND IMPROVEMENT FROM USE OF DRAINAGE-TUBE (*Service of Dr. M. O. Heydock*).—A young man, twenty-one years of years, entered Feb. 22d, 1872, with pleuro-pneumonia, which proceeded to a point where a fatal issue became imminent, when Dr. Owens gave exit to five pints of pus, by paracentesis, May 17th. In about one month it became necessary to repeat this operation, when a drainage-tube was inserted, which remained *in situ* till July 30th, when it was removed, in the fear that it might operate as a seton. But, in Setember, an aggravation of the symptoms occurred, when a third tapping of the chest was effected, and the drainage-tube reintroduced. This the patient has worn now, to his great advantage, for nearly one year and a half; and he reappeared in the hospital a few days since, in order to have the tube, which had grown weak from its long contact with pus, changed for a new one.

Can the tube now be altogether dispensed with? Six or eight weeks ago, a fragment of exfoliated bone, about one inch in length, was removed from the wound. Forced respiration is distinctly audible over almost all the surface of the chest. The discharge, at present, does not exceed two ounces daily. The cavity is thoroughly washed out with a weak solution of carbolic acid, every twenty-four hours.

CASE III.—AMPUTATION BY ESMARCH'S METHOD.—A re-amputation of the bones of a leg, which had formerly been crushed by an injury, was necessitated by the exposure of the extremities of both the tibia and fibula, at the bottom of an indolent ulcer. The stump was encircled by a rubber bandage, each successive turn of which was made to overlap its predecessor, till, at the edge of the last turn, a piece of elastic tubing was brought around the limb. The operation was as bloodless as though done in the cadaver. A small slough on the edge of one flap subsequently separated, where it lay upon a subjacent cartilage, but the result was no more than might be expected in cicatricial tissue, and did not affect the cure of the case, which was entirely satisfactory.

Some discussion of the propriety of opening a gaso-purulent abscess of the abdominal parietes ensued, in which several members participated.

Dr. Merriman gave the details of a case of pelvic cellulitis, in which, after several years of duration, there are yet alternate periods in which fœcal matter escapes from the bladder, and urinous fluid from the rectum.

Dr. Wickersham reported the case of a patient, recently under his charge, who fell upon his abdomen while at play (he was a boy of a few years of age), and subsequently suffered from dysuria. It was doubtful whether the excruciating pain which he endured, resulted from lesion of the bladder, peritoneum, or bowels. So severe was this latter symptom, that one-third of a grain of morphia was requisite to procure relief, despite his tender years. It was necessary to use the catheter twice in the day, and this was continued till an indurated mass was detected, situated, apparently, in a plane posterior to the bladder. In three or four days, fluctuation became evident, and a spontaneous exit of pus occurred at the navel. The patient made an excellent recovery.

The discussion was concluded by remarks from Drs. Merriman, Owens, Lyman, Jackson, and Hyde.

It was resolved, on motion of Dr. Owens, that every member of the Society connected with the Staff of a hospital, be added to the Committee on Clinical Reports.

It was announced that, at an early day, Dr. J. H. Etheridge would read a paper on the "Organic Hydrides;" and Dr. Jno. Bartlett would present the "Annual Report of the Section on Pathology."

The Secretary extended an invitation to the members of the Society, from the Chicago College of Pharmacy, to attend the Annual Exercises of the College, on the evening of the 10th inst.

The Society then adjourned.

